# Genetic Variability in Antioxidative and Inflammatory Pathways Modifies the Risk for PCOS and Influences Metabolic Profile of the Syndrome

**DOI:** 10.3390/metabo10110439

**Published:** 2020-10-29

**Authors:** Rok Herman, Mojca Jensterle, Andrej Janež, Katja Goričar, Vita Dolžan

**Affiliations:** 1 Faculty of Medicine, University of Ljubljana, 1000 Ljubljana, Slovenia; rokherman2@gmail.com (R.H.); mojca.jensterlesever@kclj.si (M.J.); andrej.janez@kclj.si (A.J.); 2 Department of Endocrinology, Diabetes and Metabolic Disease, University Medical Center Ljubljana, 1000 Ljubljana, Slovenia; 3 Pharmacogenetics Laboratory, Faculty of Medicine, Institute of Biochemistry, University of Ljubljana, 1000 Ljubljana, Slovenia; katja.goricar@mf.uni-lj.si

**Keywords:** polycystic ovary syndrome, genetic variability, oxidative stress, inflammation, metabolic profile

## Abstract

Polycystic ovary syndrome (PCOS) is a complex endocrine and metabolic disorder of multifactorial etiopathology likely to involve the interactions between genetics and lifestyle. Chronic inflammation and oxidative stress (OS) may participate in the pathophysiology of the syndrome. The question of the extent to which OS and inflammation are causally related to the development of the syndrome and metabolic complications remains unanswered. By our knowledge, the role of the NLR family pyrin domain containing 3 (NLRP3) inflammasome as an important trigger of inflammatory pathways and *NLRP3* and *CARD8* polymorphisms has never been addressed in PCOS yet. We conducted a case-control study conducting of total 169 Slovenian PCOS patients and 83 healthy blood donors. They were genotyped for polymorphisms in antioxidative (*SOD2* rs4880, *CAT* rs1001179, *PON1* rs854560, and rs662) and inflammatory pathways genes (*NLRP3* rs35829419, *CARD8* rs2043211, *TNF* rs1800629, *IL1B* rs1143623, and rs16944, *IL6* rs1800795) using competitive allele-specific polymerase chain reaction (PCR). Logistic regression and the Mann–Whitney test were used in the statistical analysis. *SOD2* rs4880, *CARD8* rs2043211, and *IL1B* rs16944 were associated with the risk of developing PCOS. Furthermore, the interactions between *CARD8* rs2043211 and *IL6* rs1800795 and between *IL1B* rs1143623 and *IL6* rs1800795 also significantly affected the risk for PCOS. With regard to glucose homeostasis, *CAT* rs1001179, *SOD2* rs4880, *PON1* rs854560, *NLRP3* rs35829419, and *TNF* rs1800629 were significantly associated with response to the glycemic load. Our data indicate that the genetic variability in the antioxidative and inflammatory pathways influences the development of PCOS and glucose homeostasis in PCOS patients.

## 1. Introduction

Polycystic ovary syndrome (PCOS) is the most common endocrine disorder in women in the reproductive period as it affects between 4 and 12% of the female population. It has been shown that both genetic and environmental factors play a role in PCOS development and its numerous clinical manifestations [1]. PCOS appears to have a multigenic trait, although the contributing genes remain undefined [2]. There is an increasing evidence that chronic inflammation and oxidative stress (OS) are involved in the pathogenesis; however, the question of the extent to which OS and inflammation are causally related to clinical manifestations of PCOS and the risk for development of late complications still remains unanswered.

Different types of inflammatory cytokines and chemokines are involved in PCOS [3]. When considering traditional inflammatory markers, females with PCOS were reported to have significantly higher levels of serum monocytes, lymphocytes, eosinophilic granulocytes, tumor necrosis factor α (TNF), and interleukin 6 (IL6) than controls [4]. Especially TNF seems to play a significant role in various clinical manifestations of PCOS. It is one of the most well-known inflammatory factors, and there is strong evidence that it is an essential mediator in obesity, insulin resistance (IR), and androgen expression [5]. A meta-analysis showed that TNF levels in women with PCOS were significantly higher compared to healthy controls and that high serum TNF concentration was related to IR and androgen excess but not to the body mass index (BMI) [6]. IL6, another major pro-inflammatory cytokine, has been strongly associated with IR and cardiovascular complications. A meta-analysis reported that IL6 levels were higher in women with PCOS compared to controls when matched for BMI [7]. Several studies investigated the role of interleukin-1beta (IL1B) in PCOS patients due to its alleged role in inflammatory-linked mechanisms in the ovaries. IL1B may influence prostaglandin production, mainly by its activity on cyclooxygenase-2 synthesis. It also stimulates the production of other inflammatory cytokines [8]. Lately, inflammasomes have been shown to be an important trigger of inflammatory pathways. Inflammasomes are macromolecular cytoplasmic complexes that promote the maturation and secretion of pro-inflammatory cytokines IL1B, interleukin 18, and interferon-gamma and activate nuclear factor κB. Among them, the NLR family pyrin domain containing 3 (NLRP3) inflammasome was associated with obesity and IR [9]. A component of the NLRP3 inflammasome, caspase recruitment domain-containing protein 8 (CARD8), has an inhibitory role and prevents inflammasome activation by subtle stimuli [10]. Genetic variability in *NLRP3*, but not *CARD8*, was shown to increase the risk for the development of macrovascular complications, especially myocardial infarction in patients with type 2 diabetes [11]. However, there are no data on the role of the NLRP3 inflammasome and *NLRP3* and *CARD8* polymorphisms in PCOS patients.

OS has been strongly associated with the molecular pathogenesis of PCOS [12]. In addition, many clinical manifestations of PCOS such as hyperandrogenism, obesity, and IR may contribute to the development of the local and systemic OS, which may then reciprocally worsen these metabolic abnormalities [13]. Several levels of antioxidative defense mechanisms have been established, among them antioxidative enzymes such as superoxide dismutase (SOD), catalase (CAT), and paraoxonase-1 (PON1) as well as non-enzymatic mechanisms such as antioxidants and metal-binding proteins. Antioxidative enzymes have already been reported to have an essential role in the female reproductive system and the pathogenesis of the female infertility [14]. A meta-analysis reported that the serum concentrations of several biomarkers and by-products of OS were significantly increased in PCOS patients compared with control women. However, some circulating antioxidative biomarkers were decreased in PCOS patients. A decrease was noticed in the activity of PON1, which is an antioxidant enzyme that prevents the oxidation of lipoproteins and hydrolyzes atherogenic products of oxidative lipid modification. Contrary to what was expected, this meta-analysis observed an increase in the SOD activity in PCOS patients. An increase in that potent protective enzyme that scavenges superoxide anion radical may be interpreted as a compensatory mechanism in response to the increased production of other oxidant molecules [13]. A recent study also reported a significant increase in CAT and SOD activity in PCOS patients [15].

It has been generally accepted that genetic variability in key antioxidative enzymes and inflammatory mediators can affect the individual’s ability of defense against OS and their predisposition to inflammation. The aim of our study was to investigate genetic variability in the pathways associated with OS and inflammation and its relationship with the risk of PCOS development as well as with anthropometric characteristics and glucose homeostasis in PCOS patients.

## 2. Results

### 2.1. Clinical Characteristics of PCOS Patients

Clinical, metabolic, and endocrine characteristics of PCOS patients (*n* = 169) are presented in Table 1. The median age of those patients was 30.0 (25.0–35.5). According to the Rotterdam criteria, 150 women had PCOS phenotype A, while 19 women were characterized as phenotype B due to missing ultrasound data. All of the anthropometric characteristics were above normal, and on average, our patient group can be categorized as class II obesity with a BMI of 35.8 (31.8–39.9). None of the investigated polymorphisms was associated with the anthropometric characteristics in our patient group. An oral glucose tolerance test (OGTT) was performed in only 99 patients, but 71 of them (71.1%) presented with IR, and the median homeostatic model assessment (HOMA IR) was 2.8 (1.7–5.0). In a large proportion of patients, at least one of the androgens was elevated or at the upper limit of normal, and in addition, the luteinizing hormone (LH) to follicle-stimulating hormone (FSH) ratio was more than 1, and frequently, it was more than 2, which are all common PCOS features. As with the anthropometric characteristics, no association between investigated polymorphisms and hormonal levels was observed (all *p* values > 0.05).

### 2.2. Polymorphism Frequencies in PCOS Patients and Controls and PCOS Risk

The genotype frequency distribution for the investigated polymorphisms in healthy controls and PCOS patients, odds ratio (OR) for PCOS development, and minor allele frequency (MAF) are shown in Table 2.

Subjects with at least one polymorphic *SOD2* rs4880 allele were significantly more likely to develop PCOS compared to those with a reference genotype (OR = 1.87; 95% CI = 1.03–3.37; *p* = 0.039). Carriers of two polymorphic *CARD8* rs2043211 alleles were statistically significantly less likely to develop PCOS (OR = 0.38; 95% CI = 0.18–0.84; *p* = 0.016). Carriers of one or at least one polymorphic *IL1B* rs16944 allele were also less likely to develop PCOS (OR = 0.49; 95% CI = 0.27–0.86; *p* = 0.014 and OR = 0.54; 95% Cl = 0.31–0.92; *p* = 0.024, respectively). Other investigated single nucleotide polymorphisms (SNPs) were not significantly associated with PCOS risk.

### 2.3. Interactions between CARD8, NLRP3, IL1B, and IL6 Polymorphisms and PCOS Risk

Since *CARD8*, *NLRP3*, *IL1B*, and *IL6* cooperate in the same pathways, we evaluated if interactions between polymorphisms in those genes affect PCOS risk. As it is evident in Table 3, the interaction between *CARD8* rs2043211 and *IL6* rs1800795 was significant, since the combined influence of both polymorphisms was smaller than what would be expected if we only multiplied the effects of both individual polymorphisms (OR = 0.26; 95% CI = 0.09–0.81; *p* = 0.020). On the other hand, the interaction between *IL1B* rs1143623 and *IL6* rs1800795 increased the risk for PCOS development (OR = 3.17; 95% CI = 1.04–9.65; OR = 0.042). The remaining interactions were not statistically significant.

### 2.4. Polymorphisms in Genes Related to OS and Clinical Manifestations of PCOS

Table 4 presents the data on associations of the investigated polymorphisms related to OS with clinical characteristics of PCOS patients. None of the investigated polymorphisms was associated with anthropometric characteristics in our patient group. On the other hand, *CAT* rs1001179, *SOD2* rs4880, and *PON1* rs854560 were associated with glucose or insulin levels after OGTT.

Patients with at least one polymorphic *CAT* rs1001179 allele had lower basal insulin levels than those without it (*p* = 0.001). They also had lower basal glucose levels, but the difference was not statistically significant (Table 4). Patients with at least one polymorphic *CAT* rs1001179 allele also had significantly lower HOMA IR (*p* = 0.001).

**Figure 1:**
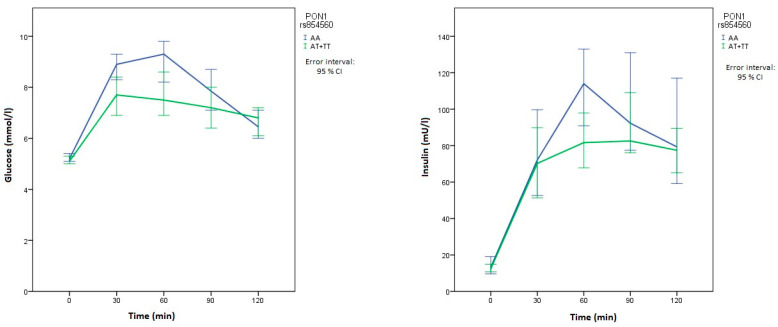
Time course of glucose and insulin levels for *PON1* rs854560. The blue line represents the reference genotype, while the green line represents patients with at least one polymorphic allele.

**Figure 2:**
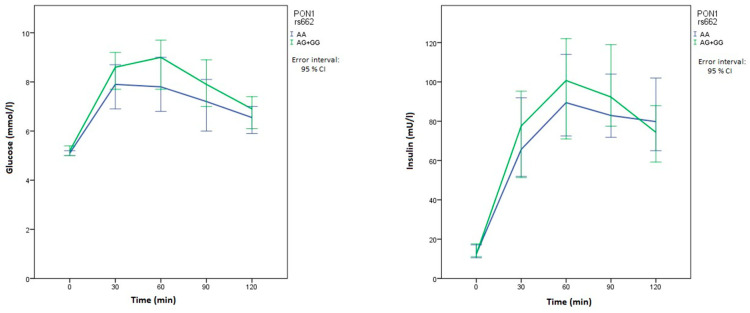
Time course of glucose and insulin levels for *PON1* rs662. The blue line represents the reference genotype, while the green line represents patients with at least one polymorphic allele.

Carriers of at least one polymorphic *SOD2* rs4880 allele had lower basal glucose levels than patients with two normal alleles (*p* = 0.038). In comparison with patients with the wild-type *PON1* rs854560 genotype, patients with at least one polymorphic allele had significantly lower glucose levels at 30 min (*p* = 0.006) and 60 min (*p* = 0.006) after the glycemic load. They also had lower glucose levels after 90 min, but the difference was not significant (Table 4). Patients with the wild-type *PON1* rs854560 genotype also had significantly higher insulin levels after 60 min (*p* = 0.024). On the other hand, *PON1* rs662 polymorphism was not significantly associated with OGTT results, although the carriers of two normal alleles had higher median glucose levels at all times compared with carriers of at least one polymorphic allele. There was an important difference in median glucose and insulin levels in their relationship with both *PON1* polymorphisms at multiple measurements in OGTT, as can be seen in graphs of the time course of glucose and insulin levels (Figure 1 and Figure 2).

### 2.5. Polymorphisms in Genes Related to Inflammation and Clinical Manifestations of PCOS

Table 5 presents the data on associations of the investigated polymorphisms related to inflammation with clinical characteristics of PCOS patients. None of the investigated polymorphisms was associated with anthropometric characteristics in our patient group. On the other hand, *NLRP3* rs35829419 and *TNF* rs1800629 were associated with glucose or insulin levels after OGTT.

**Figure 3:**
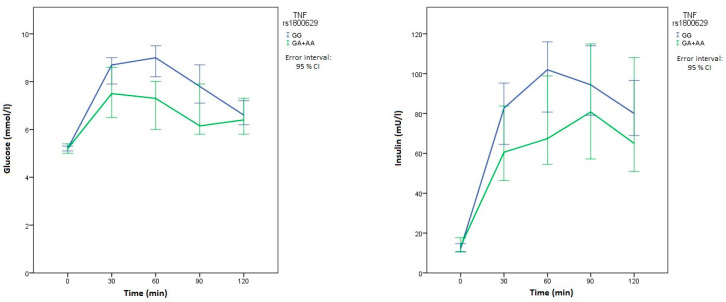
Time course of glucose and insulin concentrations for *TNF* rs1800629. The blue line represents the reference genotype, while the green line represents patients with at least one polymorphic allele.

Patients with two normal *NLRP3* rs35829419 alleles had lower glucose levels 120 min after the glycemic load than those with at least one polymorphic allele (*p* = 0.048). Carriers of at least one polymorphic *TNF* rs1800629 allele had significantly lower glucose levels 30 (*p* = 0.020), 60 (*p* = 0.007), and 90 (*p* = 0.032) min after OGTT than patients with the wild-type genotype. Furthermore, these patients also had significantly lower insulin concentrations after 60 min (*p* = 0.044). This patient group also had lower insulin concentrations at all other measurements after the glycemic load, but the differences were not statistically significant (Table 5). Since there was an important difference in median glucose and insulin levels in relationship with *TNF* polymorphism at multiple measurements of OGTT, the time course of glucose and insulin levels is presented in Figure 3.

## 3. Discussion

Our results show that polymorphisms in the investigated genes coding for antioxidant enzymes and pro-inflammatory mediators, in particular *SOD2* rs4880, *CARD8* rs2043211, and *IL1B* rs16944, modify the risk for PCOS development. Furthermore, we showed that there are significant interactions between pro-inflammatory mediators when it comes to the PCOS risk, which might imply that those mediators participate in the same pathways in PCOS pathogenesis. When we studied the effects of selected polymorphisms in patients’ clinical characteristics before the first treatment, we have observed many significant associations when it came to the results of glucose homeostasis, especially with *TNF* rs1800629, *CAT* rs1001179, *PON1* rs854560, and *NLRP3* rs35829419. These results could help us better understand the role of OS and inflammation in PCOS.

We evaluated the effect of different key polymorphisms in genes related to OS and inflammation on the risk of PCOS development and PCOS patients’ clinical characteristics due to the three main reasons. The first one is that previous studies suggested that there is an interrelationship between OS, inflammation, metabolic syndrome, and PCOS [16,17]. The second is that the question of the extent to which OS and low-grade chronic inflammation are included in PCOS pathogenesis remains unanswered. Thirdly, there is still an uncertainty to what degree inflammation and OS are causally related. Our study aimed to bring some new light on all of those questions from a different perspective.

Our results show that *CARD8* rs2043211, *SOD2* rs4880, and *IL1B* rs16944 had a significant impact on the risk for PCOS. Currently, there are no studies that would examine the relationship between CARD8 or *CARD8* polymorphisms and PCOS. It is assumed that CARD8 works as a negative regulator of the NLRP3 inflammasome and could consequently be associated with inflammation [18]. Our results show that the carriers of two polymorphic *CARD8* rs2043211 T alleles had a lower risk for PCOS. That is consistent with previous studies, since this polymorphism has already been associated with increased risk for the development of Crohn’s disease. Although the results vary in different studies, the majority of them show that the polymorphic T allele has a protective role against Crohn’s disease [19,20]. A number of other SNPs are present within the *CARD8* gene and its regulatory region, but most of the common SNPs are intronic. The ClinVar database describes no common *CARD8* genetic variants or confirmed functional variants. In the literature, several *CARD8* SNPs were investigated in only one study, while rs6509365 was evaluated more frequently [21,22,23,24,25]. This intron variant was associated with melanoma and susceptibility to tuberculosis [21,23,25]. However, in European populations, this intron SNP is in very high linkage disequilibrium with the rs2043211 investigated in our study. Still, in silico analysis suggests that some CARD8 SNPs within the regulatory region could have a putatively functional role. As they are not in linkage disequilibrium with rs2043211, it would be interesting to assess their association with PCOS in further studies.

Our results show that the polymorphic *SOD2* rs4880 allele was a significant risk factor for PCOS. Similar results were recently observed in Chinese women, where the prevalence of the polymorphic allele was significantly greater in patients with PCOS than in control group [26]. In addition, a study from 2014 compared the effect of that polymorphism on the monocyte’s response to lipopolysaccharides. When monocytes and macrophages were activated by a proper stimulus, they started to produce pro-inflammatory cytokines and reactive oxygen species. Cells expressing the normal *SOD2* allele were more quick to adapt to a more intense metabolism by upregulating cellular detoxification mechanisms, which could imply the protective role of the normal C allele against OS [27]. Previous studies also described the association of this polymorphism with the success of in vitro fertilization, where females without this polymorphic allele showed a higher pregnancy rate. In another study, that genotype also displayed a more responsive antioxidative effect with clomiphene citrate treatment than other genotypes [28]. Some studies have already examined the relationship between different polymorphisms in *IL1B* and PCOS [8,29]. In our study, carriers with at least one normal *IL1B* rs16944 allele had lower probability for PCOS development than carriers of two polymorphic alleles. Most of the other researchers focused on the same *IL1B* rs16944 polymorphism, where one study found that carriers of two normal alleles have a higher probability for PCOS, whereas the other study found out that subjects with polymorphic alleles were more likely to be obese [8,29].

Another interesting observation of our study was that interactions between *CARD8*, *NLRP3*, *IL1B*, and *IL6* modify the risk for PCOS. We noticed that the effect of *CARD8* rs2043211 and *IL1B* rs1143623 on PCOS risk varied according to *IL6* rs1800795 genotype. CARD8 most likely acts as a negative regulator of the NLRP3 inflammasome. The activation of this inflammasome leads to the cleavage of procaspase 1 into active caspase 1, which then cleaves the pro-cytokine IL1B into the mature cytokine IL1B. In addition to its essential effector functions, this cytokine promotes the expression of other cytokines, including IL6 [18,20]. Considering these associations, significant interactions between *CARD8* and *IL6* and between *IL1B* and *IL6* carry more weight and could present a starting point for further research.

When examining the impact of the selected polymorphisms on the clinical characteristics of the patients before treatment, the most statistically significant associations were found in the response to the glycemic load. The role of *TNF* rs1800629, *CAT* rs1001179, and *PON1* rs854560 was the most prominent. Carriers of the polymorphic *TNF* allele had lower glucose and insulin levels in all measurements. Our results are consistent with the generally accepted hypothesis that TNF plays an essential role in the development of IR and type 2 diabetes. Several studies have already reported increased levels of TNF mRNA in adipose tissue of obese or insulin-resistant subjects. Furthermore, elevated TNF levels were also reported in IR accompanying pathological conditions such as cachexia [29,30,31]. However, the role of different polymorphisms of this gene in IR is less well known. A study conducted in 2010 on the Tunisian population found no association between *TNF* rs1800629 polymorphism and the risk of type 2 diabetes or obesity [32]. No statistically significant effect of this polymorphism was also observed in the study conducted on an Indian population where six different *TNF* polymorphisms were studied in patients with type 2 diabetes. However, they found that carriers of polymorphic alleles in all six polymorphisms had a higher risk of developing type 2 diabetes [33]. Regarding *CAT*, we observed the effect of this polymorphism on basal glucose and insulin levels. The carriers of the polymorphic allele had lower basal glucose and insulin levels, and consequently, they had a lower HOMA IR index as well. There is a lot of evidence that genetic alterations of *CAT* and its promoter represent a risk factor for metabolic diseases. However, the influence of different polymorphisms on metabolic pathways is still not well known [34]. For *CAT* rs1001179, there are two significant studies of its impact on diabetes. A study from 2012 found that diabetic patients carrying two polymorphic alleles had an increased risk of diabetes-related complications. Furthermore, those patients had lower HDL-cholesterol levels and higher serum glucose levels [35]. The second study from Cambridge did not find a significant effect of that polymorphism on type 1 diabetes risk [36]. With *PON1* rs854560, our results show that patients with at least one polymorphic allele had decreased glucose levels at several timepoints following the glycemic load. This polymorphism has been previously associated with an increased risk of type 2 diabetes and increased risk of cardiovascular complications in diabetic patients [37]. Another meta-analysis found that this polymorphism is significantly associated with diabetic retinopathy [38]. Although *PON1* rs662 polymorphism was not significantly associated with the response to OGTT, the carriers of two normal alleles had higher median glucose levels at all times compared with carriers of at least one polymorphic allele. Among Japanese type 2 diabetes patients, carriers of the polymorphic *PON1* rs662 allele were at increased risk for developing coronary artery disease [39]. Another study on an Indian population showed that this polymorphism may serve as a biomarker to identify type 2 diabetes patients who are at risk of developing coronary artery disease [40]. These associations of studied polymorphisms on glycemic load could be essential in understanding PCOS pathogenesis, since IR plays a key role in the pathophysiology of PCOS and is associated with both ovulatory dysfunction and hyperandrogenism [41]. Our results emphasize the potential view of PCOS as a metabolic disorder, which was already indicated in recent metabolomic studies [41,42]. Several specific metabolic pathways, including protein, lipid, and carbohydrate metabolism and the TCA cycle, appear to be disturbed in PCOS [43].

The present study has some limitations. The number of patients and controls was relatively small. However, we studied relatively frequent polymorphisms, and the genotype distribution for all of them was in HWE. In addition, patients and controls were from a Slovene population that is ethnically and genetically very homogeneous. In our study, no adjustment for multiple comparisons was used, so new larger studies are needed to further validate our results. The other limitation is the difference in BMI class between patients and controls that may have affected the risk assessment for PCOS development. The investigation of the role of BMI and the interactions between BMI and genetic factors presents an opportunity for future studies that will include BMI-matched patients and controls. It is also important to be aware of the potential impact of elevated BMI in our PCOS group, because the obesity model itself can produce an alteration in the antioxidative and inflammatory profile. PCOS does not necessarily result in obesity, and now, many studies in the field subcategorize patients into lean and obese types. Comparison of our results with normal weight PCOS patients could eliminate obesity as a confounding factor in the study and clarify the influence of investigated polymorphisms in different PCOS subgroups.

## 4. Materials and Methods

Our retrospective study included 169 Slovenian PCOS patients and 83 healthy female blood donors. All patients were treated and followed up at the outpatient clinics of the Department of Endocrinology, Diabetes, and Metabolic Diseases at the University Medical Centre Ljubljana. The diagnosis was established using the Rotterdam criteria with all included patients characterized as phenotype A or B. Phenotype A was defined as the concomitant presence of hyperandrogenism, ovulatory dysfunction, and polycystic ovarian morphology (PCOM) and represents the classical form of the syndrome. Phenotype B presents as hyperandrogenism and ovulatory dysfunction, without confirmed PCOM [44]. Our diagnostic criteria were in agreement with the latest revised guidelines suggesting that ultrasound in adults may not be required if oligo-or anovulation and hyperandrogenism are present [45]. All subjects were at least 18 years old and informed of the study’s aims and provided written consent before they were enrolled in the study. Exclusion criteria were cardiovascular diseases, pregnancy, cancer, type 2 diabetes, and other diseases associated with chronic inflammation. The control group included healthy young women aged between 20 and 25 years, with normal BMI, who had normal menstrual frequency and no signs of hyperandrogenism. They also met the same exclusion criteria as the patients’ group. The study was approved by the Republic of Slovenia National Medical Ethics Committee (PCOS group—124/01/12, 2 February 2012; control group—43/02/09, 7 March 2009) and was carried out according to the Helsinki Declaration.

Anthropometric data and metabolic parameters were obtained at the beginning of the study before any treatment protocol began. Anthropometric data included height [cm], weight [kg], waist circumference [cm], BMI [kg/m^2^] and estimated mass [g], volume [cm^3^] and surface [cm^2^] of visceral adipose tissue (VAT). The estimated VAT values were measured using dual-energy X-ray absorptiometry (Hologic, Waltham, MA). The PCOM was confirmed if the following ultrasound criteria for PCOM were met: a follicle number per ovary of more than 20 follicles (2–9 mm) and/or an ovarian volume ≥10 mL using transducer frequency ≥8 MHz, or the criterian of ovarian volume ≥10 mL for older ultrasound equipment [45]. A standard oral glucose tolerance test (OGTT) was performed at the inclusion in the study with glucose and insulin levels measured before and 30, 60, 90, and 120 min after the glycemic load. Blood glucose levels were determined using a standard glucose oxidase method (Beckman Coulter Glucose Analyzer, Beckman Coulter Inc CA, USA), while the insulin levels were determined using an immunometric test (Diagnostic Systems Laboratories, Webster, Tx). For determining IR, homeostatic model assessment (HOMA IR) was calculated, and values greater than 2.0 were considered as indicative of the presence of IR. For measurements of hormone levels, the samples were collected in the morning in a fasting condition. Whenever possible, we followed the recommendation that androgens should be evaluated during the ovulatory phase, especially in the first 3–5 days of the menstrual cycle. Androstenedione and DHEAS were measured by specific double antibody RIA using 125 I-labeled hormones (Diagnostic Systems Laboratories, Webster, Tx). Total and free testosterone levels were measured by a coated tube RIA (DiaSorin, S. p. A, Salluggia, Italy and Diagnostic Products Corporation, LA, respectively). The intra-assay coefficient of variation (CV) for androstenedione ranges from 5.0 to 7.5% and the inter-assay CV ranges from 4.1 to 11.3%, the intra-assay CV for dehydroepiandrosterone sulfate (DHEAS) ranges from 4.9 to 9.8% and the inter-assay CV ranges from 7.9 to 13.0%. The intra-assay CV for free testosterone is 7.7–19.3%, and the inter-assay CV is 6.4–13.2%. The intra-assay CV for total testosterone is 5.1–16.3%, and the inter-assay CV is 7.2–24.3%.

In molecular genetic analysis, genomic DNA was isolated from peripheral blood leukocytes using the FlexiGene DNA kit (Qiagen, Hilden, Germany). Selected single nucleotide polymorphisms (SNPs) were genotyped using a fluorescent-based competitive allele-specific polymerase chain reaction (KASPar) assay (LGC Genomics, UK) or real-time PCR-based Taqman SNP genotyping assay (Applied Biosystems, Foster City, CA, USA) following the manufacturer’s instructions. In total, we genotyped four polymorphisms related to OS (*SOD2* rs4880, *CAT* rs1001179, *PON1* rs854560 and rs662) and six related to inflammation (*NLRP3* rs35829419, *CARD8* rs2043211, *IL1B* rs16944 and rs1143623, *IL6* rs1800795, *TNF* rs1800629). The genotype distribution for all investigated polymorphisms was in Hardy-Weinberg equilibrium (HWE) (all *p* > 0.05). *PON1* rs854560 and rs662 were not in linkage disequilibrium (LD) (R^2^ = 0.202). *IL1B* rs1143623 and rs16944 were slightly linked, but below the cutoff of 0.8 (R^2^ = 0.733).

In the statistical analysis, the distribution of categorical data was presented with the frequencies, while the continuous variables were presented with median and interquartile range (25–75th percentile). A Chi square test was used to assess deviation from HWE. In the analysis of PCOS risk, the wild-type genotype served as a reference, and its frequency was then compared with the frequencies of other genotypes in additive and dominant models. In *IL1B*, only the recessive model was used, as the normal T allele had lower frequency than the polymorphic C allele in the European population. In *NLRP3* rs35829419 and *TNF* rs1800629, only the dominant model was used due to the low minor allele frequency (MAF) values. In the analysis of polymorphisms influence on anthropometric characteristics and glucose homeostasis, only the dominant model was used. The differences in the distribution of categorical data and possible interactions between genotypes were determined by using logistic regression. When analyzing continuous variables, the Mann–Whitney test was used. The differences were considered statistically significant when the *p*-value was below 0.05. All statistical analyses were performed using IBM SPSS Statistics version 21.0 (IBM Corporation, Armonk, NY, USA).

## 5. Conclusions

In conclusion, the importance of OS and inflammation and their causal relationship in PCOS development have been speculated for a long time, but so far, most studies have only measured different markers of OS and inflammation in PCOS patients. Our data indicate that genetic variability in genes coding for antioxidant enzymes and inflammatory pathway mediators not only modifies the risk for PCOS but also influences the metabolic characteristics of PCOS patients. More extensive studies with age and BMI-matched control and study groups could explore the role of the selected polymorphisms in PCOS pathogenesis even further. Furthermore, better understanding of the role of genetic variability in these pathways may be of importance for everyday clinical practice through identifying patients’ groups that are at risk for PCOS development and predicting their clinical progression and their risk for metabolic complications.

## Figures and Tables

**Table 1:** Characteristics of polycystic ovary syndrome (PCOS) patients.

Characteristic	Median Value (25–75%)
Age (years)	30 (25–35.5)
**Anthropometric Characteristics**	
Body mass (kg)	100.1 (85.1–111.2)
BMI (kg/m^2^)	35.8 (31.8–39.9)
Waist circumference (cm)	112 (102–121.8)
VAT mass (g)	760 (580.3–965.0)
VAT volume (cm^3^)	819.5 (627.8–1038.5)
VAT surface (cm^2^)	157 (117–199)
**OGTT**	
Glucose 0 min OGTT (mmol/L) Reference: 3.6–6.1	5.2 (4.8–5.6)
Glucose 30 min OGTT (mmol/L)	8.3 (6.9–9.4)
Glucose 60 min OGTT (mmol/L)	8.3 (6.8–9.8)
Glucose 90 min OGTT (mmol/L)	7.6 (5.9–9.1)
Glucose 120 min OGTT (mmol/L)	6.6 (5.5–7.9)
Insulin 0 min OGTT (mU/L) Reference: 2–17.2	12.4 (7.5–20.0)
Insulin 30 min OGTT (mU/L)	71.2 (45.2–104.5)
Insulin 60 min OGTT (mU/L)	97.3 (63.3–131.5)
Insulin 90 min OGTT (mU/L)	89.9 (62.1–121)
Insulin 120 min OGTT (mU/L)	78 (53.3–123.5)
HOMA IR Reference: <2.0	2.8 (1.7–5.0)
**Endocrine Characteristics**	
DHEAS * (μmol/L) Reference: 0.95–11.67	5.8 (4.0–7.4)
Total testosteron (nmol/L) Reference: <2.53	1.8 (1.2–2.6)
Free testosteron (pmol/L) Reference: follicular phase: 1.56–11.00	7 (4.6–8.7)
SHBG* (nmol/L) Reference: 18–144	24 (18–35)
Androstenedion (nmol/L) Reference: 0.7–10.8	8.7 (6.5–11.2)
LH (IU/L) Reference: follicular phase: 1.1–11.6;	5.4 (3.0–8.7)
FSH (mIU/L) Reference: follicular phase: 2.8–11.3;	4.8 (3.8–6.6)

* DHEAS—Dehydroepiandrosterone sulfate, SHBG—Sex hormone-binding globulin.

**Table 2:** The frequencies of investigated polymorphisms in PCOS patients and controls.

Gene	SNP	Genotype	Number of Controls (%)	Number of Patients (%)	OR (95% CI)	*p*	MAF
**Genes Related to OS**							
*CAT*	rs1001179 c.-330C>T	CC	40 (49.4)	96 (57.1)	Reference		0.284
		CT	36 (44.4)	65 (38.7)	0.75 (0.43–1.30)	0.310	
		TT	5 (6.2)	7 (4.2)	0.58 (0.17–1.95)	0.381	
		CT+TT	41 (50.6)	72 (42.9)	0.73 (0.43–1.25)	0.250	
*SOD2*	rs4880 p.Ala16Val	CC	27 (32.9)	35 (20.8)	Reference		0.445
		CT	37 (45.1)	91 (54.2)	1.90 (1.01–3.57)	**0.047**	
		TT	18 (22.0)	42 (25.0)	1.80 (0.85–3.80)	0.123	
		CT+TT	55 (67.1)	133 (79.2)	1.87 (1.03–3.37)	**0.039**	
*PON1*	rs854560 p.Leu55Met	AA	30 (36.1)	75 (45.2)	Reference		0.373
		AT	44 (53.0)	71 (42.8)	0.65 (0.37–1.14)	0.130	
		TT	9 (10.8)	20 (12.0)	0.89 (0.36–2.17)	0.796	
		AT+TT	53 (63.8)	91 (54.8)	0.69 (0.40–1.18)	0.174	
*PON1*	rs662 p.Gln192Arg	AA	44 (53.0)	82 (49.1)	Reference		0.265
		AG	34 (41.0)	73 (43.7)	1.15 (0.67–1.99)	0.612	
		GG	5 (6.0)	12 (7.2)	1.29 (0.43–3.89)	0.654	
		AG+GG	39 (47.0)	85 (50.9)	1.17 (0.69–1.98)	0.560	
**Genes Related to Inflammation**							
*CARD8*	rs2043211 p.Cys10Ter	AA	30 (36.1)	78 (46.4)	Reference		0.428
		AT	35 (42.2)	72 (42.9)	0.79 (0.44–1.42)	0.431	
		TT	18 (21.7)	18 (10.7)	0.38 (0.18–0.84)	**0.016**	
		AT+TT	53 (63.9)	90 (53.6)	0.65 (0.38–1.12)	0.123	
*NLRP3*	rs35829419 p.Gln705Lys	CC	76 (91.6)	145 (86.3)	Reference		0.042
		CA	7 (8.4)	22 (13.1)	/		
		AA	0 (0.0)	1 (0.6)			
		CA+AA	7 (8.4)	23 (13.7)	1.72 (0.71–4.20)	0.231	
*TNF*	rs1800629 c.-308 G>A	GG	61 (77.2)	120 (73.6)	Reference		0.120
		GA	17 (21.5)	41 (25.2)	/		
		AA	1 (1.3)	2 (1.2)			
		GA+AA	18 (22.8)	43 (26.4)	1.21 (0.65–2.28)	0.546	
*IL1B*	rs1143623 c.-1560G>C	GG	43 (51.8)	105 (62.1)	Reference		0.271
		GC	35 (42.2)	51 (30.2)	0.60 (0.34–1.04)	0.070	
		CC	5 (6.0)	13 (7.7)	1.06 (0.36–3.17)	0.910	
		GC+CC	40 (48.2)	64 (37.9)	0.66 (0.39–1.11)	0.119	
*IL1B*	rs16944 c.-598T>C	TT	9 (11.1)	20 (11.9)	0.76 (0.31–1.84)	0.541	0.630 *
		TC	42 (51.9)	60 (35.7)	0.49 (0.27–0.86)	**0.014**	
		CC	30 (37.0)	88 (52.4)	Reference		
		TC+CC	72 (88.9)	148 (88.1)	0.54 (0.31–0.92)	**0.024**	
*IL6*	rs1800795 c.-174G>C	GG	34 (41.0)	60 (35.7)	Reference		0.373
		GC	36 (43.4)	87 (51.8)	1.37 (0.77–2.43)	0.282	
		CC	13 (15.7)	21 (12.5)	0.92 (0.41–2.06)	0.831	
		GC+CC	49 (59.1)	108 (64.3)	1.25 (0.73–2.14)	0.419	

* frequency of polymorphic allele; statistically significant *p*-values are bolded.

**Table 3:** The effect of interactions between *CARD8, NLRP3, IL1B*, and *IL6* polymorphisms on PCOS risk.

Interaction	OR (95% CI)	*p*
*CARD8* rs2043211 and *IL1B* rs1143623	1.27 (0.43–3.80)	0.666
*CARD8* rs2043211 and *IL1B* rs16944	0.67 (0.22–2.06)	0.480
*CARD8* rs2043211 and *IL6* rs1800795	0.26 (0.09–0.81)	**0.020**
*NLRP3* rs35829419 and *IL1B* rs1143623	0.71 (0.31–1.62)	0.412
*NLRP3* rs35829419 and *IL1B* rs16944	1.21 (0.18–8.28)	0.843
*NLRP3* rs35829419 and *IL6* rs1800795	1.41 (0.23–8.58)	0.707
*IL1B* rs1143623 and *IL6* rs1800795	3.17 (1.04–9.65)	**0.042**
*IL1B* rs16944 and *IL6* rs1800795	0.44 (0.14–1.37)	0.158

statistically significant *p*-values are bolded.

**Table 4:** Effects of polymorphisms in genes related to oxidative stress (OS) on PCOS patients’ clinical manifestations.

Characteristic	Genotype *	*CAT* rs1001179	*p*	*SOD2* rs4880	*p*	*PON1* rs854560	*p*	*PON1* rs662	*p*
		Median Value (25–75%)		Median Value (25–75%)		Median Value (25–75%)		Median Value (25–75%)	
**Anthropometric Characteristics**									
Body mass (kg)	XX	101.7 (86.9–111.8)	0.371	100 (85.8–105.7)	0.365	100.2 (82–110.1)	0.198	101 (89.9–112.1)	0.147
	Xx+xx	97.9 (83–110.4)		100.6 (84.9–112)		100 (89.8–112)		100 (82–110)	
BMI (kg/m^2^)	XX	36.9 (32–41)	0.106	35.5 (31.4–39.9)	0.598	36 (30.8–39.9)	0.631	36.6 (33.2–40.4)	0.071
	Xx+xx	35.5 (31.2–38.6)		36 (31.8–39.9)		35.8 (32.7–39.8)		34.6 (30.8–39.1)	
Waist circumference (cm)	XX	112.5 (103.3–123.8)	0.220	108 (102.5–118.8)	0.416	112 (99–124.5)	0.728	112.5 (102.8–122.6)	0.448
	Xx+xx	108.5 (100–119.5)		113 (101.5–123)		111.5 (103.8–121)		110 (101–121)	
VAT mass (g)	XX	797 (614–990)	0.227	852 (746–990)	0.162	793 (616–968)	0.490	694.5 (561.3–914)	0.195
	Xx+xx	685 (535–879)		744 (565–959.5)		756 (523–970.5)		810 (651–971)	
VAT volume (cm^3^)	XX	857 (664–1070)	0.266	921 (807–1070)	0.141	857 (666–1046)	0.421	751 (606.5–987.5)	0.231
	Xx+xx	740 (578–950)		781.5 (610.8–1018)		810.5 (565.5–1044.5)		874 (704–1050)	
VAT surface (cm^2^)	XX	165 (127.3–205)	0.107	177 (155–205)	0.126	162 (127.3–198.3)	0.534	145 (116–201)	0.487
	Xx+xx	140 (110–177)		148 (116–194)		155.5 (108.8–200.5)		167 (124–199)	
**OGTT**									
Glucose 0 min OGTT (mmol/L)	XX	5.2 (4.9–5.6)	0.051	5.4 (5.1–5.7)	**0.038**	5.2 (4.9–5.6)	0.558	5.1 (4.8–5.4)	0.353
	Xx+xx	5.1 (4.8–5.4)		5.1 (4.8–5.5)		5.1 (4.8–5.5)		5.2 (4.9–5.7)	
Glucose 30 min OGTT (mmol/L)	XX	8.2 (6.9–9.4)	0.821	8.2 (6.7–9.3)	0.474	8.9 (7.9–9.5)	**0.006**	7.9 (6.5–9.3)	0.063
	Xx+xx	8.4 (6.9–9.4)		8.4 (7–9.4)		7.7 (6.5–9.3)		8.6 (7.5–10.2)	
Glucose 60 min OGTT (mmol/L)	XX	8.7 (6.8–9.9)	0.886	8.1 (6.7–9.7)	0.661	9.3 (8–10.6)	**0.006**	7.8 (6.3–9.7)	0.069
	Xx+xx	8.3 (7.1–9.7)		8.5 (6.8–10.1)		7.5 (6–9.7)		9 (7.2–11.2)	
Glucose 90 min OGTT (mmol/L)	XX	7.6 (5.7–9)	0.583	7.4 (5.9–8.9)	0.681	7.9 (7–9.2)	0.166	7.2 (5.7–8.7)	0.066
	Xx+xx	7.7 (6.3–9.3)		7.8 (6.2–9.1)		7.2 (5.7–9.1)		7.9 (6.5–9.6)	
Glucose 120 min OGTT (mmol/L)	XX	6.8 (5.6–8)	0.158	6.6 (5.8–7.8)	0.677	6.5 (5.6–7.5)	0.660	6.6 (5.5–7.6)	0.359
	Xx+xx	6.3 (5.4–7.6)		6.6 (5.5–7.9)		6.8 (5.5–7.9)		6.9 (5.5–8.1)	
Insulin 0 min OGTT (mU/L)	XX	15.2 (11.1–21.6)	**0.001**	11.8 (9.3–21.1)	0.822	13.2 (6.7–22.3)	0.628	12.5 (7.7–20.1)	0.873
	Xx+xx	9.3 (5.9–17.1)		12.5 (7.3–20)		12.4 (7.9–19.8)		12.4 (7.5–20)	
Insulin 30 min OGTT (mU/L)	XX	74.9 (48.1–102)	0.413	67.6 (43.5–95.9)	0.869	72.2 (45.7–110.3)	0.598	65.7 (46.2–106)	0.961
	Xx+xx	68.4 (42.8–108.8)		71.3 (45.1–108.8)		70.3 (44–104.3)		77.6 (43.2–106.3)	
Insulin 60 min OGTT (mU/L)	XX	87.2 (64.7–125.5)	0.833	78.8 (56.6–138.3)	0.878	114 (76.1–154.8)	**0.024**	89.5 (63.7–130)	0.716
	Xx+xx	99.2 (61.5–136.8)		97.5 (63.2–129.8)		81.7 (56.3–116)		100.7 (59.7–139.5)	
Insulin 90 min OGTT (mU/L)	XX	91.2 (67.1–119.5)	0.836	82.9 (67.1–118.5)	0.811	92.3 (69.6–154)	0.327	82.9 (62.2–117.3)	0.426
	Xx+xx	86.6 (57.7–139.5)		90.6 (59.2–125)		82.6 (60.5–118.3)		92.3 (61.5–145)	
Insulin 120 min OGTT (mU/L)	XX	87 (56.9–136)	0.065	79.8 (54.8–138.3)	0.869	79.3 (50.8–133.8)	0.607	79.8 (51.3–137)	0.598
	Xx+xx	66.1 (49.3–94.3)		77.5 (52–121.8)		77.5 (53.7–121.3)		74.4 (54.2–124)	
HOMA IR	XX	3.3 (2.5–5.4)	**0.001**	3 (2.2–5.2)	0.607	2.9 (1.6–5.4)	0.605	2.8 (1.7–4.6)	0.792
	Xx+xx	2.3 (1.3–3.5)		2.8 (1.7–4.8)		2.8 (1.8–4.5)		2.8 (1.8–5.1)	

* X—major (common) allele; x—minor (variant) allele; statistically significant *p*-values are bolded.

**Table 5:** Effects of polymorphisms in genes related to inflammation on PCOS patients’ clinical manifestations.

Characteristic	Genotype *	*CARD8* rs2043211		*NLRP3* rs35829419		*TNF* rs1800629		*IL1B* rs1143623		IL1B rs16944 **		*IL6* rs1800795	
		Median Value (25–75%)	*p*	Median Value (25–75%)	*p*	Median Value (25–75%)	*p*	Median Value (25–75%)	*p*	Median Value (25–75%)	*p*	Median Value (25–75%)	*p*
**Anthropometric Characteristics**													
Body mass (kg)	XX	101 (89.7–113.1)	0.226	100 (85–112)	0.990	101 (84.6–112.2)	0.801	99.9 (86.6–110.6)	0.776	100.5 (83–111.5)	0.702	101(86.6–112)	0.659
	Xx+xx	98 (84.8–110.6)		101(85.6–106)		99.7(88.9–108.5)		101.7(83.8–112.5)		100 (87.4–110.7)		100(84.5–110.4)	
BMI (kg/m^2^)	XX	36.6 (32.1–40.3)	0.272	35.8 (31.7–40)	0.551	36 (31.8–39.9)	0.828	36 (31.8–40)	0.995	35.5 (31.1–39.5)	0.346	35.7 (31.9–40)	0.821
	Xx+xx	35.5 (31.6–39.8)		37.5 (32.2–39.8)		34.6 (32.1–39.9)		35.7 (31.7–39.9)		37 (31.9–40.3)		36 (31.1–39.7)	
Waist circumference (cm)	XX	112 (102–127)	0.527	110.3(101.3–121)	0.097	112 (102–123)	0.567	110.8(100.8–121)	0.183	112(103–123)	0.469	112.5(102–120.5)	0.696
	Xx+xx	111.5(101.8–119.5)		117 (109–125)		110 (102–119)		114 (104–128.8)		111(100–121)		111 (101–122.8)	
VAT mass (g)	XX	759 (573.5–989)	0.669	758 (580.3–959.5)	0.634	797 (614–988)	0.111	755 (588.5–898)	0.542	710(541.5–979.5)	0.363	698.5(521.5–949)	0.533
	Xx+xx	761 (581–937)		804 (562–1015)		699(514.8–852)		795(567.8–1003.5)		805.5(611–952.5)		761 (612–969.5)	
VAT volume (cm^3^)	XX	818 (620–1069)	0.768	816 (627.8–1018)	0.613	862 (664–1068)	0.073	810.5(636.8–954.5)	0.503	768(585.5–1059)	0.403	745 (563.5–975.5)	0.423
	Xx+xx	823 (628.5–1013)		869 (607–1097)		747(556.3–909.8)		859.5(613.5–1085)		843.5(661–1003.5)		823 (662–1048)	
VAT surface (cm^2^)	XX	155 (116–199.5)	0.864	156 (117–194)	0.568	165 (124–201)	0.202	153.5(115.5–179.8)	0.272	152.5(113.8–205)	0.718	144 (110.5–204.5)	0.739
	Xx+xx	158.5(122.3–199.5)		167 (116–211)		144 (108–177)		165 (118.5–211)		156.5(124.8–187.5)		157 (125.5–200)	
**OGTT**													
Glucose 0 min OGTT (mmol/L)	XX	5.1 (4.8–5.6)	0.847	5.2 (4.8–5.6)	0.568	5.2 (4.9–5.6)	0.762	5.1 (4.8–5.6)	0.547	5.1 (4.9–5.5)	0.616	5.2 (4.9–5.6)	0.572
	Xx+xx	5.2 (4.9–5.6)		5.2 (4.9–5.7)		5.2 (4.7–5.7)		5.2 (4.9–5.5)		5.2 (4.8–5.7)		5.1 (4.8–5.6)	
Glucose 30 min OGTT (mmol/L)	XX	8.4 (6.9–9.3)	0.833	8.4 (6.9–9.3)	0.620	8.7 (7.3–10.1)	**0.020**	8.4 (6.7–9.3)	0.552	7.9 (7–10)	0.840	8.1 (6.9–9.5)	0.492
	Xx+xx	8.3 (7.1–9.6)		8.1 (6.9–10.7)		7.5 (6.3–9)		8.1 (7.1–10.5)		8.6 (6.9–9.3)		8.5 (7–9.4)	
Glucose 60 min OGTT (mmol/L)	XX	8.5 (6.9–9.7)	0.997	8.3 (6.8–9.7)	0.396	9 (7.1–10)	**0.007**	8.2 (6.7–9.6)	0.236	8.1 (6.8–11.1)	0.808	8.3 (6.9–9.5)	0.724
	Xx+xx	8.3 (6.7–10)		8.8 (6.7–12)		7.3 (5.9–8.4)		8.7 (6.9–11.5)		8.6 (6.7–9.7)		8.5 (6.5–10.5)	
Glucose 90 min OGTT (mmol/L)	XX	7.6 (5.7–9.4)	0.862	7.4 (5.9–8.9)	0.227	7.8 (6.6–9.1)	**0.032**	7.4 (5.8–9.1)	0.457	7.8 (6–9.1)	0.896	7.7 (6.7–9)	0.849
	Xx+xx	7.6 (6.2–8.7)		8.8 (6.4–9.6)		6.2 (5.7–8.1)		7.9 (6.6–9)		7.4 (5.8–9.1)		7.5 (5.8–9.4)	
Glucose 120 min OGTT (mmol/L)	XX	6.9 (5.5–7.9)	0.396	6.5 (5.5–7.7)	**0.048**	6.6 (5.5–7.9)	0.742	6.9 (5.5–7.8)	0.995	6.6 (5.3–7.7)	0.708	7.2 (5.9–8.2)	0.091
	Xx+xx	6.4 (5.5–7.8)		7.6 (6.2–9.3)		6.4 (5.5–7.8)		6.6 (5.5–7.9)		6.9 (5.6–8)		6.3 (5.4–7.5)	
Insulin 0 min OGTT (mU/L)	XX	12.5 (9.2–21.1)	0.303	11.8 (7–19.7)	0.116	12.2 (7.1–20.1)	0.611	11.4 (6.9–19.6)	0.076	12.6 (7.7–20.2)	0.829	12 (6.1–20.8)	0.520
	Xx+xx	12.4 (7–19.1)		14.3 (11.3–24.2)		13.4 (9.3–20)		14.2 (10.6–22.5)		11.8 (7.5–19.8)		12.4 (8.6–20)	
Insulin 30 min OGTT (mU/L)	XX	64 (40.8–99.9)	0.090	67.8 (44.8–101.3)	0.420	82.7 (45.3–104)	0.305	70.2 (45.2–102.9)	0.728	73.2 (43.4–112)	0.886	81.3 (41.6–113)	0.755
	Xx+xx	81.3 (47.9–112)		79.9 (61.8–110)		60.6 (43.4–110)		73.2 (44.8–111.5)		71.1 (49.4–99.2)		68.1 (46.3–100)	
Insulin 60 min OGTT (mU/L)	XX	80.7 (63.3–138.5)	0.768	94.1 (60.3–129.3)	0.242	102 (72.5–137)	**0.044**	98.4 (63.2–136.8)	0.650	85.5 (54.2–128)	0.251	104 (75.4–140.3)	0.268
	Xx+xx	99.2 (62–128.8)		102 (76.9–147)		67.4 (54.2–111)		87.2 (60.8–127.5)		99.4 (66.8–138)		85.5 (61.8–127)	
Insulin 90 min OGTT (mU/L)	XX	81.2 (61.3–118.5)	0.722	84.2 (59.2–118.3)	0.092	94.4 (62.4–126)	0.160	90.6 (64.2–128.8)	0.974	79.9 (58.4–122)	0.647	104.1 (59.1–150.5)	0.470
	Xx+xx	101.6 (64.2–124.5)		115 (80.3–148)		80.8 (56.1–115)		79.9 (60.6–119)		91.2 (71.4–125)		85.8 (62.4–119)	
Insulin 120 min OGTT (mU/L)	XX	77.5 (56.4–117.5)	0.985	72.6 (51.6–113.5)	0.056	80 (61.3–128)	0.138	72.6 (49.4–124)	0.344	80 (51.8–122)	0.678	80 (53–146)	0.694
	Xx+xx	80.3 (48.2–125.5)		122 (77.8–162)		65 (44.2–123.5)		80.3 (55.1–124)		73.1 (54.4–127.5)		77.5 (52.9–120)	
HOMA IR	XX	2.8 (2.2–5.1)	0.313	2.8 (1.6–4.9)	0.104	2.8 (1.7–5.1)	0.664	2.7 (1.6–4.8)	0.079	3 (1.9–4.9)	0.869	3 (1.4–4.8)	0.655
	Xx+xx	2.8 (1.6–4.8)		3.3 (2.6–6.3)		3.1 (2.1–4.8)		3.2 (2.5–5.2)		2.8 (1.7–5)		2.8 (2.1–5.1)	

* X—major (common) allele; x—minor (variant) allele; ** with *IL1B* rs16944 we compared XX+Xx vs xx; statistically significant *p*-values are bolded.
